# Evaluating active roles of community health workers in accelerating universal access to health services for malaria in Palawan, the Philippines

**DOI:** 10.1186/s41182-016-0008-7

**Published:** 2016-04-10

**Authors:** Emilie Louise Akiko Matsumoto-Takahashi, Shigeyuki Kano

**Affiliations:** Department of Tropical Medicine and Malaria, Research Institute, National Center for Global Health and Medicine, 1-21-1 Toyama, Shinjuku-ku, Tokyo, 162-8655 Japan

**Keywords:** Universal health access, Malaria, Community health workers, Microscopist, Palawan, The Philippines

## Abstract

**Background:**

Palawan is the most malaria-endemic province in the Philippines. In an effort to confront malaria in areas with limited healthcare facilities, microscopists (community health workers) have been trained to diagnose malaria since 1999.

**Methods:**

We reviewed the epidemiological data and related literature which analyzed the achievements of the microscopists and their tasks in order to propose a strategy to strengthen community-based malaria control.

**Results:**

The epidemiological data showed that there had been a drastic decrease in malaria morbidity and mortality throughout the province following the launch of the strategy. Microscopists clearly enhanced the feasibility of early diagnosis and treatment throughout the province. However, it remained necessary to implement anti-malarial measures focusing on children under 5 years of age who live in the southern region of the province. The analysis of our published papers also enabled us to propose a new strategy to enhance activities by microscopists to raise malaria awareness in their respective communities.

**Conclusions:**

These low-cost activities are expected to strengthen the preventive measures implemented by the residents and to drive more people to seek appropriate treatment. Consequently, this new strategy could accelerate the efforts to eliminate malaria in the province of Palawan that will be adopted in the WHO’s Global Technical Strategy for Malaria 2016–2030, in order to achieve the Sustainable Development Goals by 2030.

## Background

Malaria is one of the most important parasitic infections in humans. In 2013, it accounted for approximately 584,000 deaths (range 367,000–755,000) worldwide [1]. Although malaria was once prevalent throughout much of the inhabited world, it has been eliminated in roughly half of the world’s countries and now is mostly restricted to rural tropical regions [2]. From 2000 to 2013, the number of malaria infections dropped by 26 % from 173 million to 128 million, while the worldwide mortality rate decreased by 47 % [1]. This decrease occurred as a result of substantial increases in donor funding, improved control, and increased enthusiasm for malaria elimination [3, 4].

Improving the availability of effective health services has been an essential strategy for malaria elimination in all of the endemic regions [1]. At present, malaria mostly remains in remote and rural regions where essential preventive measures are delivered to the populations that need them the most by poor health systems with insufficient human resources [5, 6]. Thus, the targeting of residents in these areas, including socially vulnerable groups such as ethnic minorities and migrants, is a key to malaria control [7, 8].

To overcome these challenges in many malaria-endemic regions, the validity and usefulness of strategies that utilize community health workers (CHWs) has been proposed [9, 10]. According to the World Health Organization (WHO), CHWs should meet the following criteria: be members of the communities where they work; be selected by the communities; be answerable to the communities for their activities; be supported by health systems, but not necessarily a part of their organization; and have shorter training than professional workers [11]. In rural areas, where there is a recognized paucity of human capacity and health systems, the utilization of CHWs is a potentially inexpensive, effective, and sustainable approach for bringing malaria diagnosis and treatment closer to households [11].

Although CHWs, such as barangay health workers (BHWs) or government-trained health volunteers, have been operating in rural villages in the Philippines since 1981, their involvement in malaria control has not been satisfactory [12]. This has largely been due to inadequate training, insufficient logistical support, poorly sustained motivational schemes, and a lack of community support.

With this background, a community-based malaria control program was established in Palawan, one of the most malaria-endemic provinces in the Philippines, in 1999 [13–16]. The program, which was called *Kilusan Ligtas Malaria* (KLM) (Tagalog: Movement Against Malaria), involved training 344 CHWs as malaria microscopists (one microscopist per malaria-endemic village). The microscopists diagnose malaria in febrile patients via microscopy and prescribe first-line anti-malarial drugs to malaria-infected patients. Microscopists also implement community awareness-raising activities that are aimed at preventing the transmission of malaria among their patients and their patients’ families. Since this program started, diagnostic techniques that allow for the early diagnosis of malaria have been extended to the entire province. This community-based malaria control program was partly maintained with the aid of the Japan International Cooperation Agency and the ongoing Global Fund Project through the Pilipinas Shell Foundation, Inc.

To accelerate the efforts to eliminate malaria in Palawan, this community-based malaria control program must be evaluated. The present study also aimed to find out the value of the capacity for microscopy and active roles of CHWs by reviewing the epidemiological data in Palawan and the related literature. The findings may also be used to enhance the performance of CHWs in other settings.

## Methods

### Study area

Palawan is the most malaria-endemic province in the Philippines. Eighteen out of the 23 legislated municipalities and the capital city of Puerto Princesa are considered to be malarious [13–16]. The capital city is located in the center of this oblong island province and divides the island into the northern and southern regions. The island is mostly covered by tropical rainforests, which limit the residents’ access to affordable healthcare facilities. In 2010, the population of Palawan was 1,025,800. The residents lived in 367 villages in 23 municipalities [17]. The ethnicities of the residents include Tagalog (the predominant ethnic group in the Philippines), Cuyunon, Hiligaynon, Palawan, Cebuano, Ilocano, Bisaya, Kagayanan, and Tagbanwa.

### Data collection

We reviewed the epidemiological data and related literature which analyzed the achievements of the microscopists and their tasks. Available epidemiological data of malaria morbidity and mortality in Palawan were collected in the Provincial Health Office of Palawan and KLM main office in Puerto Princesa City, Palawan, the Philippines. Besides, we searched and reviewed related articles in PubMed which had been published since the earliest date up to December 2015.

## Results

### Malaria in the Philippines

Malaria has existed in the Philippines for centuries, and several malaria control strategies have been implemented [18–20]. When the Spanish arrived and started to occupy the Philippines in the 16th century, the country was largely covered with extensive forests, which the local people avoided due to the risk of infection [18]. Forest exploitation using forced local labor contributed to several malaria epidemics and the persistence of malaria within the Philippines. After the Spanish-American War in 1898, the United States occupied the Philippines and programs were implemented to minimize the spread of several diseases including malaria, cholera, smallpox, dysentery, and tuberculosis. In 1931, there were approximately 20,000 deaths due to malaria per year and the strategy for malaria control was to examine blood smears by microscope, to treat cases with quinine alone or with plasmochin, and to use screens and bed nets [19]. The start of World War II saw an increase in malaria morbidity and mortality throughout the Philippines.

After the end of World War II, malaria-vector control was achieved by dichloro-diphenyl-trichloroethane (DDT) spraying in the province of Mindanao, an island in the southeast of the Philippines [18, 20]. DDT spraying proved effective, and the spraying program was expanded to cover the rest of the Philippines in 1955; thereafter, there was a dramatic decrease in malaria morbidity. However, spraying alone was by no means a perfect tool for eliminating malaria on the islands.

### Evidence-based diagnoses and treatment by microscopists

**Fig. 1:**
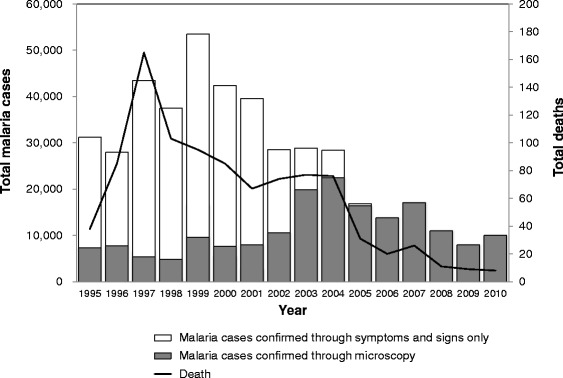
The malaria trends in Palawan. The figure was created by the authors based on the data of the Provincial Health Report, 1995–2010 [13–16]

KLM initiated its activities in 1999 [21]. Between 1990 and 1997, the incidence of malaria and malaria-related deaths actually increased in Palawan until 1997 and then began to decrease year by year (Fig. 1). After 2006, the total number of blood smears inspected remained at around 90,000 and the annual blood examination rate (the number of blood smears examined in a year/total population * 100) remained at around 10; however, there was a yearly decrease in the slide positivity rate (SPR) [13–16]. The number of deaths caused by malaria also began to decrease from a peak of 170 in 1997 to 9 in 2009.

In 1995, before KLM began its program of training microscopists, 76.1 % of malaria diagnoses were made through symptoms and signs (Fig. 1). The proportion of evidence-based diagnoses by the microscopists increased after 1999. Consequently, in 2005, only 500 out of 16,885 patients were diagnosed based on symptoms and signs. Since 2006, all of the patients have reportedly been microscopically diagnosed.

### Activities of community awareness-raising for prevention

**Fig. 2:**
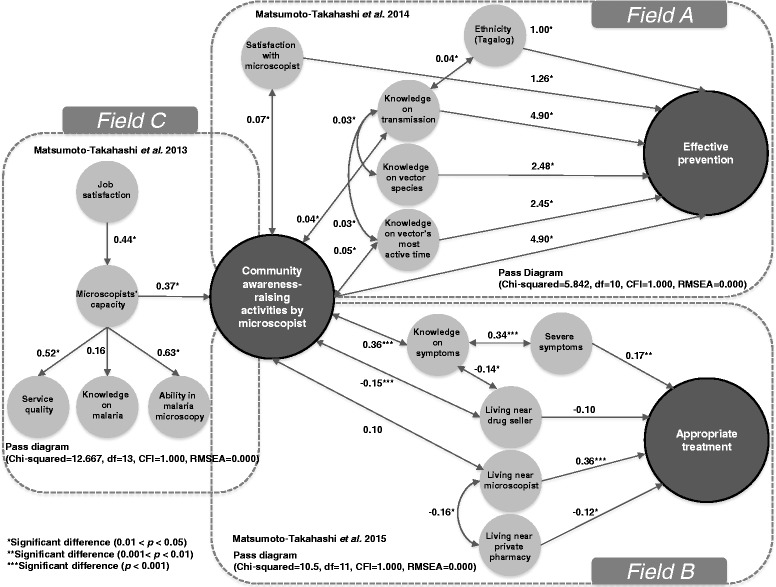
The determinants of community awareness-raising activities by microscopists, appropriate treatment, and effective prevention

As summarized in Fig. 2, the community awareness-raising activities of the microscopists strengthened the effective prevention practices implemented by the residents of Palawan and increased the likelihood that they would seek appropriate treatment. Community awareness-raising activities that inform residents about transmission, vector species, and the times that vector species are most active were expected to be effective in strengthening malaria prevention practices (Fig. 2, Field A) [22]. Knowledge on transmission was statistically proven to be the most important factor in this regard (pass coefficient, 4.90). To increase the likelihood that residents would seek appropriate treatment, community awareness-raising activities that sought to improve knowledge on malaria symptoms were important to make inhabitants more aware of their symptoms; this led to improved self-triage (Fig. 2, Field B) [23]. The study suggested that improving the residents’ recognition of malaria symptoms and making them aware of nearby microscopists would likely be the keys to accelerating universal access to effective malaria treatment in Palawan.

These activities would be enhanced by additional follow-up interventions to improve service quality and microscopic usage ability (Fig. 2, Field C) [24]. High-capacity microscopists could be expected to offer high service quality (active detection, diagnosis and treatment, prescription of anti-malarial, and follow-up) and to show a high level of ability in malaria microscopy (preparation and documentation, slide preparation and observation, safe handling and disposal, and knowledge on the morphology of infected red blood cells).

In addition, these activities should be intensively practiced to strengthen the preventive measures implemented by indigenous residents and people who travel to mountain areas (Fig. 2, Field A) [22, 23]. In Palawan, most of the ethno-linguistic groups live in the forests, while other residents must stay in the forest for subsistence (e.g., farming or mining). Because malaria transmission mainly occurs in the forests or adjacent areas, malaria control among those groups is the key to eliminating malaria [25–27].

### Overall morbidity and mortality

The trends in the leading causes of morbidity and mortality in Palawan from 2005 to 2009 are shown in Tables 1 and 2. Prior to 2005, most of the leading causes of morbidity were infectious diseases such as malaria, respiratory infections, and urinary tract infections [14]. There were at least 170 malaria-related deaths in 1997 (Fig. 1).

**Table 1:** Trends in the leading causes of morbidity in Palawan from 2005 to 2009

Causes of morbidity	2005			2006			2007			2008			2009		
	Top 10	Number	Rate^a^	Top 10	Number	Rate^a^	Top 10	Number	Rate^a^	Top 10	Number	Rate^a^	Top 10	Number	Rate^a^
URTI/ARI	1st	28,011	4303	1st	27,959	4201	1st	19,217	2392	1st	24,030	3457	1st	38,857	4964
Influenza	2nd	17,085	2625	2nd	16,091	2418	2nd	16,266	1477	2nd	16,719	2405	2nd	13,528	1728
Malaria^b^	3rd	10,334	1588	3rd	12,640	1899	3rd	10,048	973	5th	5865	844	4th	6558	838
Diarrhea	4th	7038	1081	4th	8932	1342	4th	6617	831	4th	7061	1016	7th	3655	467
Bronchitis	5th	4800	737	5th	7908	1188	6th	4382	521	3rd	8303	1195	6th	3738	477
Hypertension	6th	3317	510	6th	4843	728	7th	3540	502	7th	3000	432	3rd	7335	937
Urinary tract infection	7th	2700	415	7th	2856	429	8th	3417	185	6th	5671	816	5th	4483	573
Pneumonia	8th	2260	347	8th	1470	221	5th	5652	644	8th	2994	431	10th	2471	316
Anemia	9th	1447	222	10th	858	129									
Pulmonary tuberculosis	10th	1029	158	9th	949	143	10th	971	127						
Skin disease							9th	1261	143						
Gastritis										9th	1402	202	9th	2066	264
Accident/wound/injury										10th	1130	163	8th	2834	362

The table was created by the author based on the annual provincial health report of the province of Palawan [15, 16]

*Abbreviations*: *URTI* upper respiratory tract infection, *ARI* acute respiratory infection

^a^Rate per 100,000 population

^b^The number does not match that in Table 1 due to differences in the methods of data collection (the data for this table was only collected in provincial and private hospitals)

**Table 2:** Trends in the leading causes of mortality in Palawan from 2005 to 2009

Causes of mortality	2005			2006			2007			2008			2009		
	Top 10	Number	Rate^a^	Top 10	Number	Rate^a^	Top 10	Number	Rate^a^	Top 10	Number	Rate^a^	Top 10	Number	Rate^a^
Pneumonia	1st	239	37.0	1st	224	33.7	1st	313	46.0	1st	285	41.0	1st	337	43.1
Hypertension	2nd	155	24.0	5th	101	15.2	7th	78	11.5				4th	108	13.8
Pulmonary tuberculosis	3rd	128	20.0	3rd	122	18.3	4th	163	24.0	4th	97	14.0	6th	99	12.7
Unspecified natural cause	4th	123	19.0	9th	60	9.0	3rd	181	26.7	10th	57	8.2			
Cancer	5th	105	16.0	4th	105	15.7	5th	137	20.1	2nd	107	15.4	2nd	123	15.8
Diarrhea	6th	95	15.0	8th	63	9.5	6th	125	18.4				8th	72	9.2
Myocardial infarction	7th	83	13.0	2nd	147	22.1				8th	59	8.5			
Accident/wound/injury	8th	67	10.0												
Stillbirth	9th	56	9.0							6th	63	9.1			
Myocarditis	10th	54	8.0				8th	76	11.2	9th	59	8.5	9th	69	8.8
Congestive heart failure				6th	87	13.1									
Renal failure				7th	80	12.0	9th	72	10.6	7th	63	9.1	7th	77	9.8
Peptic ulcer							10th	54	8.0						
Cardiovascular disease							2nd	295	43.4	3rd	103	14.9	5th	101	12.9
Cerebrovascular accident										5th	85	12.2	3rd	111	14.2
Diabetes mellitus													10th	64	8.2

The table was created by the author based on the annual provincial health report of the province of Palawan [15, 16]

^a^Rate per 100,000 population

From 2005, malaria was no longer a leading cause of death in the province, although the morbidity of malaria is still high (Tables 1, 2), and lifestyle-related diseases, such as hypertension and cardiovascular disease, were coming to be common causes of death among Palawan residents.

Coming up in 2009, total number of confirmed malaria cases were 7606 including *Plasmodium falciparum* (73.1 %), *Plasmodium vivax* (24.0 %), and *Plasmodium malariae* (1.7 %); the rest were mixed infections (data not shown). Table 3 precisely shows that there were regional differences in the annual parasite index (API). More specifically, while the human populations of the southern and northern regions were almost the same (334,392 and 330,879, respectively) in 2009, 2.4 times as many smears were inspected in the southern region. Furthermore, the SPR and API were, respectively, 5.3 times and 12.9 times higher than in the northern region. Regional differences were also found in the proportion of malaria infections in children younger than 5 years of age (Table 3). In the northern region, they accounted for 11.1 % of malaria infections. In contrast, 19.0 % of the malaria infections in the southern region were in children under 5 years of age. Thus, in the southern region, a greater percentage and a greater number of malaria infections occur in children under 5 years of age.

**Table 3:** The population distribution and malaria incidence per region (2009)

Region	Population	Smear	Confirmed malaria cases	*P. falciparum*	<5^a^ (%)	ABER	SPR	API
Total	872,390	89,290	7606	73.1 %	17.3	10.3	8.52	8.72
Northern region	334,392	23,626	488	66.0 %	11.1	7.07	2.07	1.46
Puerto Princesa City	207,119	8776	880	63.5 %	8.4	4.24	10.0	4.25
Southern region	330,879	56,888	6238	75.0 %	19.0	17.2	11.0	18.9

*Abbreviations*: *ABER* annual blood examination rate, *SPR* slide positivity rate, *API* annual parasite index

^a^The percentage of malaria patients who were younger than 5 years of age

## Discussion

Angluben et al. introduced KLM as a strategy with a focus on social mobilization in the implementation of malaria prevention and control [21]. Since the launch of KLM, microscopists had enhanced the feasibility of early diagnosis and treatment throughout the province. The achievements of KLM were reflected in the total number of malaria smears that were performed, the increase in microscopic confirmations of malaria, and the decline in clinical diagnoses between 2000 and 2006.

A novel malaria elimination strategy which aims to strengthen community awareness-raising activities by microscopists, with a focus on children under 5 years of age in the southern region of the province, indigenous people, and people who have to stay in the forest, is required. The strategy should follow a simple, effective, and affordable approach to promote the implementation of effective malaria preventive measures and to encourage residents to seek appropriate treatment. Consequently, this new strategy could accelerate the efforts to eliminate malaria in the province of Palawan, which will be adopted in the WHO’s Global Technical Strategy for Malaria 2016–2030 [28], with an aim of achieving the Sustainable Development Goals by 2030 [29].

Improvements to diagnosis and treatment alone might not be sufficient to combat malaria in the southern region of the province of Palawan. Other strategies such as health promotion in schools and mother and infant medical checkups should be considered. Consideration is also needed for the geographic characteristics of the southern region which are responsible for the high rate of malaria.

As is the case in many developing nations, Palawan is facing a changing pattern of diseases. Morbidity and mortality due to infectious diseases are decreasing, while the incidence of lifestyle-related diseases or cancer is increasing. The time has come to consider whether microscopists should be given additional tasks or whether the tasks that they perform should be changed. Since Palawan has limited health facilities and human resources, task-shifting strategies that extend the mission of trained microscopists to fight these diseases should be considered.

## Conclusion

The present study proposed a new strategy to enhance activities by microscopists to raise malaria awareness in their respective communities. These activities are expected to strengthen the preventive measures implemented by the residents and to drive more people to seek appropriate treatment. Consequently, this new strategy could accelerate the efforts to eliminate malaria in the province of Palawan, the Philippines.

### Ethics approval and consent to participate

The present study was approved by the Research Ethics Committee of the University of Tokyo (no. 3001) and upheld by the Provincial Health Office of Palawan. Before the epidemiological data collections, we asked informants to sign a consent form after being informed about the study purpose and told them that they could withdraw from the study during the data collection or even afterwards.

## Abbreviations

ABER: annual blood examination rate (number of blood smears examined in a year/total population * 100)
API: annual parasite index (number of malaria patients per year/total population * 1000)
BHWs: barangay (village) health workers
CHWs: community health workers
DDT: dichloro-diphenyl-trichloroethane
KLM: *Kilusan Ligtas Malaria* (Tagalog: Movement Against Malaria)
SPR: slide positivity rate (number of positive slides/total blood smears * 100)
WHO: World Health Organization

## References

[1] WHO | World Malaria Report 2014. 2014. http://www.who.int/malaria/publications/world_malaria_report_2014/report/en/. Accessed 26 Nov 2015.

[2] White NJ, Pukrittayakamee S, Hien TT, Faiz MA, Mokuolu OA (2014). Malaria. Lancet.

[3] Alonso PL, Brown G, Arevalo-Herrera M, Binka F, Chitnis C (2011). A research agenda to underpin malaria eradication. PLoS Med.

[4] Feachem RG, Phillips AA, Targett GA, Snow RW (2010). Call to action: priorities for malaria elimination. Lancet.

[5] World Bank. World Development Report 2004: making services work for poor people. 2004. https://openknowledge.worldbank.org/handle/10986/5986. Accessed 26 Nov 2015.

[6] Mathauer I, Imhoff I (2006). Health worker motivation in Africa: the role of non-financial incentives and human resource management tools. Hum Resour Health.

[7] Moore SJ, Min X, Hill N, Jones C, Zaixing Z (2008). Border malaria in China: knowledge and use of personal protection by minority populations and implications for malaria control: a questionnaire-based survey. BMC Public Health.

[8] Khamsiriwatchara A, Wangroongsarb P, Thwing J, Eliades J, Satimai W (2011). Respondent-driven sampling on the Thailand-Cambodia border. I. Can malaria cases be contained in mobile migrant workers?. Malar J.

[9] Christopher JB, Le May A, Lewin S, Ross DA (2011). Thirty years after Alma-Ata: a systematic review of the impact of community health workers delivering curative interventions against malaria, pneumonia and diarrhoea on child mortality and morbidity in sub-Saharan Africa. Hum Resour Health.

[10] Smith Paintain L, Willey B, Kedenge S, Sharkey A, Kim J (2014). Community health workers and stand-alone or integrated case management of malaria: a systematic literature review. Am J Trop Med Hyg.

[11] Lehmann U, Sanders D. Community health workers: what do we know about them? 2007. http://www.who.int/hrh/documents/community_health_workers.pdf. Accessed 26 Nov 2015.

[12] Lariosa TR (1992). The role of community health workers in malaria control in the Philippines. Southeast Asian J Trop Med Public Health.

[13] Provincial Health Office of Palawan (1995). Provincial health report, 1995.

[14] Provincial Health Office of Palawan (2000). Provincial health report, 2000.

[15] Provincial Health Office of Palawan (2005). Provincial health report, 2005.

[16] Provincial Health Office of Palawan (2010). Provincial health report, 2010.

[17] National Statistical Coordination Board (2012). Population of the Philippines census years 1799 to 2010.

[18] Tongol-Rivera P, Kano S, Tongol-Rivera P (2005). Milestones in the history of malaria research and its control in the Philippines. Asian parasitology.

[19] The Rockefeller Foundation (1931). The Rockefeller Foundation Annual Report.

[20] Trigg PI, Kondrachine AV (1998). Commentary: malaria control in the 1990s. Bull World Health Organ.

[21] Angluben RU, Trudeau MR, Kano S, Tongol-Rivera P (2008). Kilusan Ligtas Malaria: advancing social mobilization towards sustainable malaria control in the province of Palawan, the Philippines. Trop Med Health.

[22] Matsumoto-Takahashi EL, Tongol-Rivera P, Villacorte EA, Angluben RU, Yasuoka J (2014). Determining the impact of community awareness-raising activities on the prevention of malaria transmission in Palawan, the Philippines. Parasitol Int.

[23] Matsumoto-Takahashi EL, Tongol-Rivera P, Villacorte EA, Angluben RU, Jimba M (2015). Patient knowledge on malaria symptoms is a key to promoting universal access of patients to effective malaria treatment in Palawan, the Philippines. PLoS One.

[24] Matsumoto-Takahashi EL, Tongol-Rivera P, Villacorte EA, Angluben RU, Yasuoka J (2013). Determining the active role of microscopists in community awareness-raising activities for malaria prevention: a cross-sectional study in Palawan, the Philippines. Malar J.

[25] Incardona S, Vong S, Chiv L, Lim P, Nhem S (2007). Large-scale malaria survey in Cambodia: novel insights on species distribution and risk factors. Malar J.

[26] Singh N, Mishra AK, Shukla MM, Chand SK (2003). Forest malaria in Chhindwara, Madhya Pradesh, central India: a case study in a tribal community. Am J Trop Med Hyg.

[27] Dysoley L, Kaneko A, Eto H, Mita T, Socheat D (2008). Changing patterns of forest malaria among the mobile adult male population in Chumkiri District, Cambodia. Acta Trop.

[28] WHO. Global Technical Strategy for Malaria 2016–2030. 2015. http://www.who.int/malaria/areas/global_technical_strategy/en/. Accessed 26 Nov 2015.

[29] United Nations Sustainable Development Knowledge Platform. Transforming our world: the 2030 agenda for sustainable development. 2015. https://sustainabledevelopment.un.org/post2015/transformingourworld. Accessed 26 Nov 2015.

